# Membrane Hsp70-supported cell-to-cell connections via tunneling nanotubes revealed by live-cell STED nanoscopy

**DOI:** 10.1007/s12192-018-00958-w

**Published:** 2019-01-10

**Authors:** Judith Reindl, Maxim Shevtsov, Günther Dollinger, Stefan Stangl, Gabriele Multhoff

**Affiliations:** 1 Universität der Bundeswehr München, Werner-Heisenberg-Weg 39, 85577 Neubiberg, Germany; 2 Center for Translational Cancer Research (TranslaTUM), Klinikum rechts der Isar, Technischen Universität München (TUM), Ismaningerstrasse 22, 81675 Munich, Germany; 3 Institute of Cytology of the Russian Academy of Sciences (RAS), Tikhoretsky ave. 4, St. Petersburg, 194064 Russia; 4 Pavlov First Saint Petersburg State Medical University, L’va Tolstogo str. 6/8, St. Petersburg, 197022 Russia; 5 Polenov Russian Scientific Research Institute of Neurosurgery, Mayakovskogo str. 12, St. Petersburg, 191104 Russia

**Keywords:** Membrane-bound Hsp70, Live-cell STED, Tunneling nanotubes, Cell-to-cell connection, Cellular networks

## Abstract

Heat shock protein Hsp70 (Hsp70) is found on the cell surface of a large variety of human and mouse tumor cell types including U87, GL261 glioblastoma, and 4T1 mammary carcinoma cells. We studied the role of membrane-bound Hsp70 (mHsp70) in the formation of cell-to-cell connections via tunneling nanotubes (TNTs) using live-cell STED nanoscopy. This technique allows the visualization of microstructures in the 100-nm range in the living cells. We could show that the presence of tumor-derived mHsp70 in TNTs with a diameter ranging from 120 to 140 nm predominantly originates from cholesterol-rich-microdomains containing the lipid compound globoyltriaosylceramide (Gb3). Under non-stress conditions, Hsp70 and Gb3 are structurally clustered in the membrane of TNTs of tumor cells that showed tumor type specific variations in the amount of cell-to-cell connection networks. Furthermore depletion of cholesterol and ionizing radiation as a stress factor results in a complete loss of Hsp70-containing TNTs.

**Electronic supplementary material:**

The online version of this article (10.1007/s12192-018-00958-w) contains supplementary material, which is available to authorized users.

## Introduction

Heat shock proteins (HSPs) with a molecular weight of approximately 70 kDa constitute to a large family of highly conserved proteins that are involved in protein homeostasis, cell proliferation, differentiation, and carcinogenesis (Shevtsov and Multhoff 2016; Hartl and Hayer-Hartl 2002; Mayer and Bukau 2005). Apart from its cytosolic overexpression, the highly stress-inducible member Hsp70 (HSPA1A) is frequently found on the cell membrane in a large variety of different tumor types (Thorsteinsdottir et al. 2017; Multhoff et al. 1995). The presence of Hsp70 on the plasma membrane of tumor cells was determined by global profiling of membrane proteins (Shin et al. 2003) and flow cytometry using cmHsp70.1 monoclonal antibody (Stangl et al. 2011; Multhoff et al. 1995) that specifically detects a conformational epitope of Hsp70 in the context with lipids. Analysis of the lipid composition of membrane Hsp70-positive (mHsp70) tumor cells revealed that globoyltriaosylceramide (Gb3), which is residing in cholesterol-rich microdomains of tumor but not normal cells, serves as a potential binding partner for mHsp70 (Gehrmann et al. 2008; Simons and Toomre 2000; Nimmervoll et al. 2015). Apart from its documented immunological relevance as a tumor-specific biomarker for antibody- (Stangl et al. 2001) and NK cell-based-targeted therapies (Specht et al. 2015), the role of mHsp70 is poorly understood. The formation of tunneling membrane nanotubes (TNTs) that enable connections between living neighboring tumor cells under non-stress conditions could be determined as a novel function for mHsp70 by live-cell STED nanoscopy.

Tunneling nanotubes (TNTs) that are supposed to mediate unidirectional, actin-dependent vesicle and organelle transport (Marzo et al. 2012) were firstly described by Rustom and co-workers (Rustom et al. 2004) in rat brain tumor cells. Apart from cancer cells, TNTs were also identified in prion- or virally infected cells (Ariazi et al. 2017; Sherer 2013), healthy endothelial and immune cells, and neurons to support cell-cell communication (Marzo et al. 2012; Watkins and Salter 2005; Koyanagi et al. 2005; Önfelt et al. 2006). Calcium signaling (Sowinski et al. 2008), mitochondria (Vignais et al. 2017), mRNA (Haimovich et al. 2017), and exosomal transports (Dupont et al. 2018) have been found to be mediated by TNTs. Additionally, therapy resistance of malignant gliomas has been associated with the presence of TNTs (Weil et al. 2017), and large tubular networks have been found to be involved in tumor cell proliferation and invasion (Osswald et al. 2015).

The length of TNTs varies from a few micrometers up to several cell diameters, and the diameter of TNTs ranges between 50 and 200 nm (Rustom et al. 2004). Many publications report on stress-inducible formations of TNTs with a main transport direction from a stressed, TNT-forming cell to a non-stressed recipient cell (Marzo et al. 2012).

Due to the small diameters of TNTs, most structural analysis has been performed by SEM or TEM electron microscopy (Lou et al. 2017) with fixed, non-viable cells. Therefore, only static but not dynamic cellular processes can be monitored. Live-cell imaging using conventional optical microscopy is limited in resolution by the abbe limit (~ 200–250 nm) and therefore detailed structural analysis of the composition of nanotubes is impossible.

Herein, we report on live-cell super-resolution, gated STED nanoscopy (Hell and Wichmann 1994; Vicidomini et al. 2011; Bottanelli et al. 2016) with a sub-diffraction resolution of < 100 nm which allows a more detailed analysis of substructures of TNTs in viable tumor cells under physiological culture conditions.

Human and mouse glioblastoma (U87, GL261) and mouse mammary (4T1) carcinoma cells show a highly structured, dotted pattern of mHsp70 in Gb3-containing cholesterol-rich microdomains after staining with fluorescence-labeled cmHsp70.1 and Gb3 antibodies, as determined by conventional light microscopy (Gehrmann et al. 2015). To study the role of mHsp70 in the formation of cell-to-cell connections via nanotubes, we performed high-resolution live-cell STED nanoscopy. By quantitative size measurements and network analysis, a detailed insight in the structural and functional clustering of mHsp70 and Gb3 in TNTs could be determined in different cancer cell lines under physiological culture conditions to get a deeper insight in the tumor cell communication.

## Methods

### Cell lines

Human U87 (ATCC, HTB-14) glioma, mouse GL261 (ATCC, CRL-1887) glioma, and mouse 4T1 (ATCC, CRL-2539) mammary carcinoma cell lines were grown at 37 °C in RPMI-1640 cell medium supplemented with 10% fetal bovine serum (FBS), 2 mM l-glutamine and antibiotics (100 units/ml Penicillin G and 100 μg/ml streptomycin). Cell lines were regularly tested negative for mycoplasma contamination. Cells were passaged twice a week, and single-cell suspensions were derived by short-term (less than 1 min) treatment with 0.25% (*w*/*v*) trypsin, 0.53 mM EDTA.

### Flow cytometry

The expression of the membrane-bound Hsp70 on the tumor cells was determined by flow cytometry using either the FITC-conjugated cmHsp70.1 mAb (IgG1; multimmune GmbH), which is directed against the extracellular-exposed sequence of membrane Hsp70. Briefly, after incubation of viable cells (0.3 × 10^6^ cells) with the primary antibodies for 30 min at 4 °C and following two washing steps, 7-AAD viable cells were analyzed using a FACSCalibur flow cytometer (BD Biosciences). An isotype-matched (IgG_1_) control antibody was used to determine non-specific binding to cells.

### Methyl-β-cyclodextrin treatment

Ten millimolars of the cholesterol-depleting agent methyl-β-cyclodextrin **(**MbC) which is known to be non-lethal was added to the cells. Directly after adding image acquisition with a rate of 1 image per 2 min was started.

### Immunofluorescence staining

Cells (1 × 10^6^ cells/ml) were allowed to settle on non-coated glass dishes (IBIDI, Munich, Germany) and stained with cmHsp70.1-FITC mAb (1:250) (multimmune, Munich, Germany) for 30 min and then imaged using a TCS SP8 STED × 3 microscope (Leica, Germany). Isotype-matched IgG_1_ antibodies conjugated to FITC (Sigma, USA) were used as a control. For the visualization of the plasma membrane, PKH and CellVue® Fluorescent Cell Linker Kits (Sigma-Aldrich, USA) providing fluorescence labelling of live cells were applied according to manufacturer’s protocol. For the detection of the globoyltriaosylceramide (Gb3), Alexa Fluor 555–conjugated cholera toxin B subunit (Molecular Probes Europe BV) was used at a dilution of 1:10.

### STED microscopy, image processing, and depiction

The glass dishes were placed on the TCS SP8 STED × 3 microscope, which was temperature-controlled to 23 °C, as described before (Reindl et al. 2015) to avoid drift throughout the image acquisition and to ensure refractive index match between the glass slide, cells, and immersion medium. Images were acquired using × 100 objective (Leica HCX PL APO × 100/1.4 Oil). FITC labelling was imaged using 495 nm excitation laser and 592-nm STED depletion laser. The detection range was 500–538 nm. Alexa 555 staining was imaged using 550-nm excitation and 660-nm depletion with a detection range from 575 to 630 nm. Gating in both channels was from 0.3 to 6 ns; no line averaging and accumulation were performed. All samples were acquired in *z*-stacks with a distance of 250 nm and a pixel size of 50 nm where each *z*-slice was imaged in both color channels before moving to the next slice. *Z*-stacks were deconvolved using the Huygens software (SVI). Resolution was measured as the full width at half maximum of the smallest labeled spots and was 97 ± 5 nm. All images show maximum projections.

### Sample size determination and errors

The imaging for network quantification and Hsp70 thickness measurements was performed twice for 4T1 and GL261 and three times for U87. The membrane measurement was performed only in 4T1 cells and replicated once, as the lipid raft measurements for U87. In this study, the single cells are considered to be independent samples. This is also supported by the fact that the variances are equal in the experiment replications, which allowed for pooling of data for each cell line. Cell numbers for each experiment are depicted in Table 1. The standard deviation for the string thickness measurement is approx. *σ* = 20 *nm* in the worst case (GL261). The sample size, i.e., the minimum number of analyzed cells, is calculated as $n={\left(\frac{2\times c\times \sigma \times t}{L}\right)}^2$, with the width of the confidence interval *L* and the *t* and c factors (Kreyszig 1974) . The confidence interval of 90% leads to *c* = 1.645 and *t* = 1.337, which has to be used as small sample sizes are taken. A confidence interval of *L* < 30 nm as 1/6 of the string thickness leads to a sample size of *n* = 9 and *n* = 10, respectively. Therefore, the used sample sizes of each experiment are supposed to be sufficient. For thickness measurements, the standard error of the mean (SEM) was used.

**Table 1:** Cell numbers of the single experiments

Cell line	Experiment 1	Experiment 2	Experiment 3	Experiment 4	total
4T1	43	–	12	–	55
GL261	23	10	–	–	33
U87	32	14	–	35	81

Experiments 1 and 2 show the results of a staining with cmHsp70.1 mAb, experiment 3 that of a double staining using cmHsp70.1 mAb and PKH, and experiment 4 that of a double staining with cmHsp70.1 mAb and Gb3 antibody

For network quantification, the experiments were pooled and the error occurs due to counting and is calculated according to the Poisson statistics as $\frac{1}{\sqrt{n}}$.

## Results

### Clustering of mHSP70 at TNTs

STED imaging was performed in two to three independent experiments with a total number of 55 4T1, 33 GL261, and 81 U87 tumor cells. The sample size was sufficient for statistical analysis, as described in the “Methods” section.

**Fig. 1:**
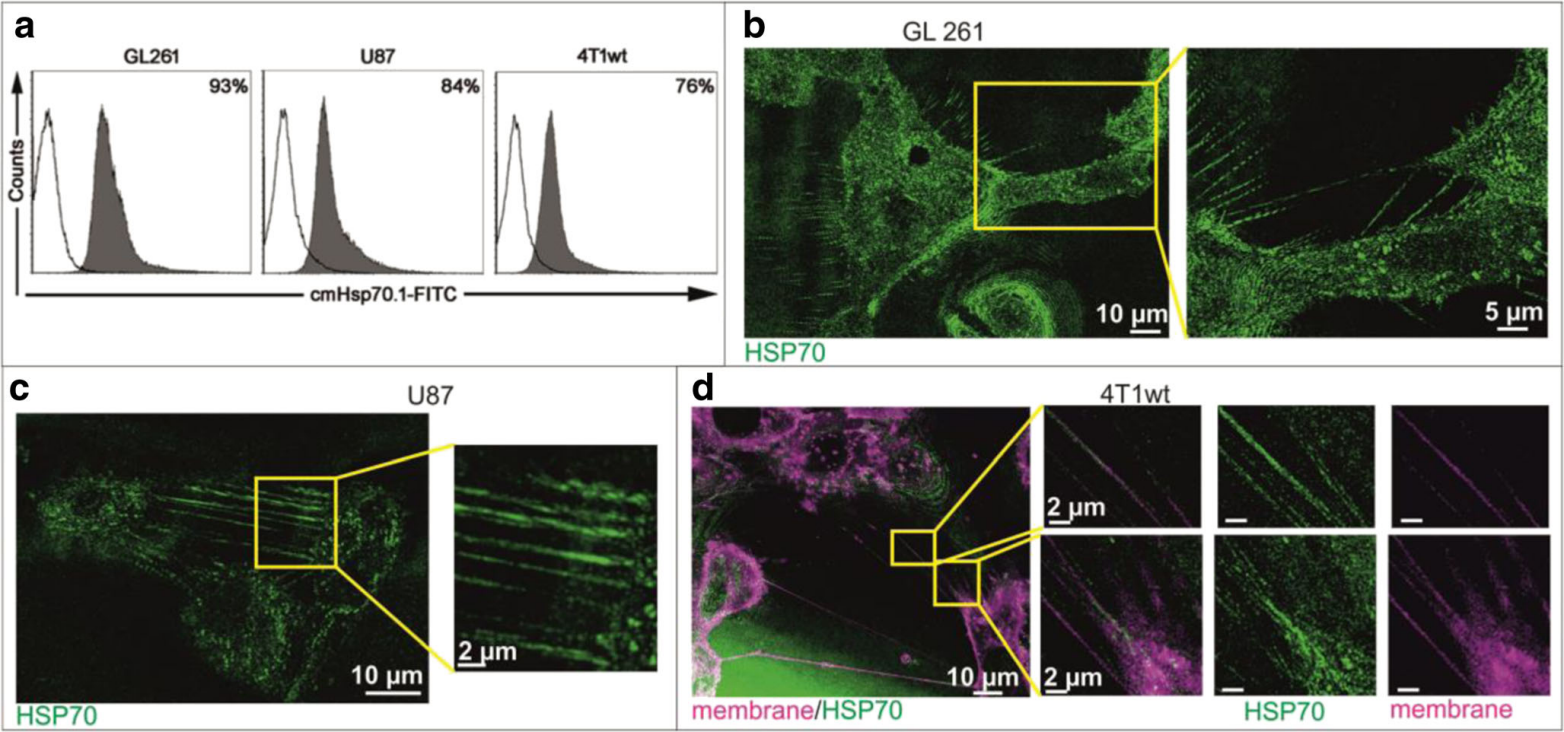
Representative flow cytometric analysis of the mHsp70 expression on GL261, U87, and 4T1 tumor cells (**a**). The proportion of positively stained cells is indicated in each graph. Hsp70 (green) is localized on the plasma membrane and in TNTs of GL261 (**b**), U87 (**c**), and 4T1 (**d**) tumor cells that are connecting neighboring tumor cells. Membranes of the tumor cells are delineated by co-staining with the PKH tracking dye (magenta) (d)

For visualization of mHsp70 in GL261, U87, and 4T1 tumor cells, the fluorescein isothiocyanate (FITC)–conjugated monoclonal antibody (mAb) cmHsp70.1 was used for analysis. This antibody recognizes an epitope of the C-terminal domain of Hsp70 that is exposed to the extracellular milieu (Stangl et al. 2011) of viable tumor cells. Binding affinities of cmHsp70.1 mAb to Hsp70 as determined in solution using microscale thermophoresis analysis revealed K_D_ values in lower nanomolar range (Stangl et al. 2018). All three tumor cell lines GL261, U87, and 4T1 were strongly positive for mHsp70 as determined by flow cytometry (Fig. 1a) and showed a typical dotted staining pattern of mHsp70 (green) on the plasma membrane, as determined by immunofluorescence studies. In addition to the membrane localization, Hsp70 was found in strings that are connecting neighboring GL261 (Fig. 1b), U87 (Fig. 1c), and 4T1 (Fig. 1d) tumor cells.

**Fig. 2:**
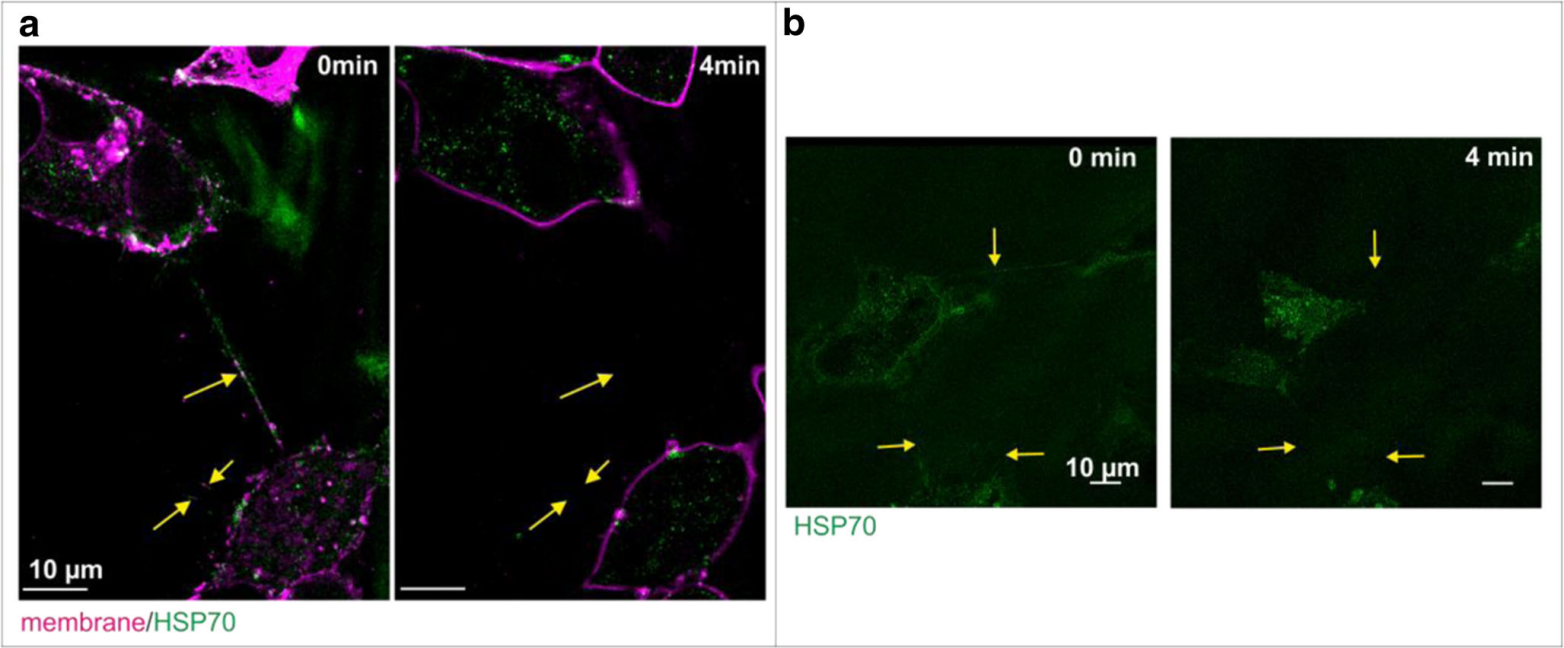
Treatment with methyl-β-cyclodextrin (MbC) results in a depletion of cholesterol, loss of Hsp70 from the plasma membrane, and a dissociation of TNTs in U87 (**a**) and GL261 cells (**b**) within 4 min. The dissociation of TNTs upon MbC treatment is more rapid in U87 compared to GL261 tumor cells

In contrast to cmHsp70.1 mAb, a FITC-conjugated IgG_1_ isotope-matched control antibody did not exhibit any specific staining of TNTs in GL261 tumor cells (Supplementary Figure 1). Presently, studies are ongoing to test a FITC-labeled Hsp70-reactive peptide (TPP) (Stangl et al. 2014) instead of cmHsp70.1 mAb for staining TNTs. Co-staining of viable 4T1 cells with the membrane tracking dye PKH (Spötl et al. 1995) (magenta) and FITC-labeled cmHsp70.1 mAb (green) confirms that Hsp70 is localized in the plasma membrane and in nanotubes derived thereof (Fig. 1d). A one-to-one correlation of Hsp70 and membrane stained tunneling nanotubes (TNTs) is shown in Figs. 1d and 2a. This correlation was determined by scoring all connecting nanotubes in a total of 35 U87 and 12 4T1 cells that show TNTs with both staining reagents.

Treatment of U87 and GL261 cells with a non-lethal concentration of methyl-β-cyclodextrin (MbC, 10 mM) which can deplete cholesterol from the lipid bilayer and thereby dissociates cholesterol-richmicrodomains results in a loss of Hsp70 from lipid rafts and dissociates Hsp70-containing TNTs that mediate cell-to-cell communications. In U87 cells, 4 min after incubation with a non-lethal dose of MbC, Hsp70 completely disappeared from the plasma membrane and Hsp70-containing nanotubes dissociated (Fig. 2a). As shown in the same figure, the relocation of mHsp70 from the membrane to the interior of the cell and the depletion of the TNTs are much faster than the disintegration of the outer cellular membrane. Similar results are observed in GL261 cells with a slightly slower kinetic (Fig. 2b).

### Size measurements of Hsp70-, PKH-, and Gb3-stained TNTs

The length of Hsp70-containing nanotubes ranges from a few micrometers up to > 100 μm which is equivalent to several cell diameters. The diameter of TNTs was determined by using the autocorrelation function, as previously described by Reindl et al. (2017) in detail. Briefly, the autocorrelation function (ACF) for each tube is determined. As the intensity distribution in the short dimension of the TNTs can be approximated by a Gaussian function, the full width at half maximum of the ACF directly gives the thickness of the TNT multiplied by factor of $\sqrt{2}$. The results of these measurements summarized in Table 2 show similar diameters for Hsp70-containing TNTs in 4T1 (126 ± 5 nm, SEM) and U87 (137 ± 14 nm, SEM) cells, and significantly larger diameters (*p* = 0.04, one-sided, unpaired *t* test) in GL261 cells (241 ± 9 nm, SEM). All measured diameters are larger than the resolution of 97 ± 5 nm.

**Table 2:** Diameters of Hsp70-, PKH-, and Gb3-stained TNTs in 4T1, GL261, and U87 cell lines

Cell line	Staining	Diameter (nm)
4T1	Hsp70	126 ± 5
GL261	Hsp70	241 ± 9
U87	Hsp70	137 ± 14
4T1	PKH	168 ± 4
U87	Gb3	136 ± 13

The resolution in this assay was 97 ± 5 nm. The data represent mean values and the standard error of the mean (SEM)

**Fig. 3:**
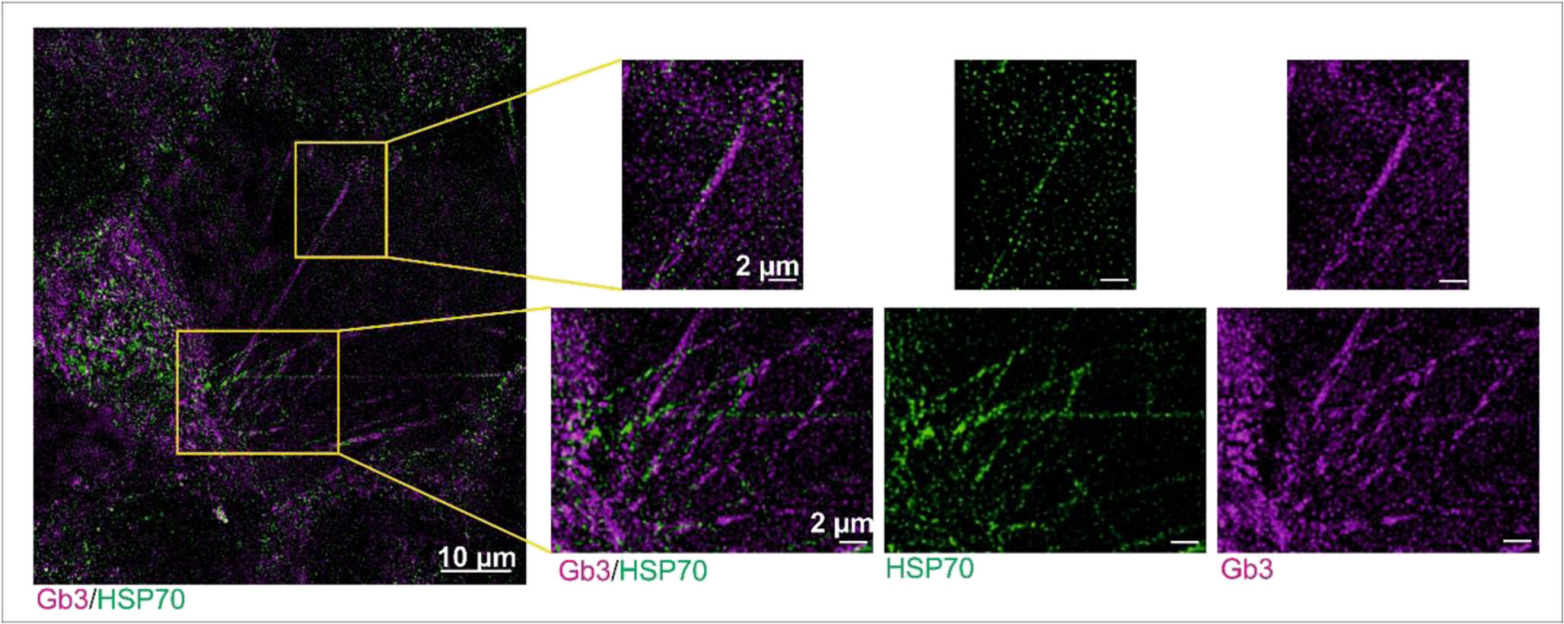
U87 cells co-stained with cmHsp70.1 (green) and Gb3 (magenta) antibodies show that both markers are in close proximity within the nanotubes. At a resolution of 100 nm, Hsp70 and Gb3 are not co-localizing along the string

The diameter of PKH-stained TNTs of 4T1 cells (168 ± 4 nm (SEM)) was comparable to that of the Hsp70-stained TNTs. A more detailed view to the staining pattern of PKH- and cmHsp70.1 mAb-stained TNTs revealed no detailed correlation within a size regime of 100 nm, as visible in the inserts in Fig. 1d, where nearly no white signals that indicate an overlap between green and magenta is visible in the tubes consisting of Hsp70 and PKH. The staining pattern of lipid raft (Gb3)- and Hsp70-stained TNTs in U87 cells (Fig. 3) exhibited identical sizes and a similar clustering of both markers in close proximity, within the TNTs (insert in Fig. 3).

### Model of HSP70 and Gb3 clustering in TNTs

**Fig. 4:**
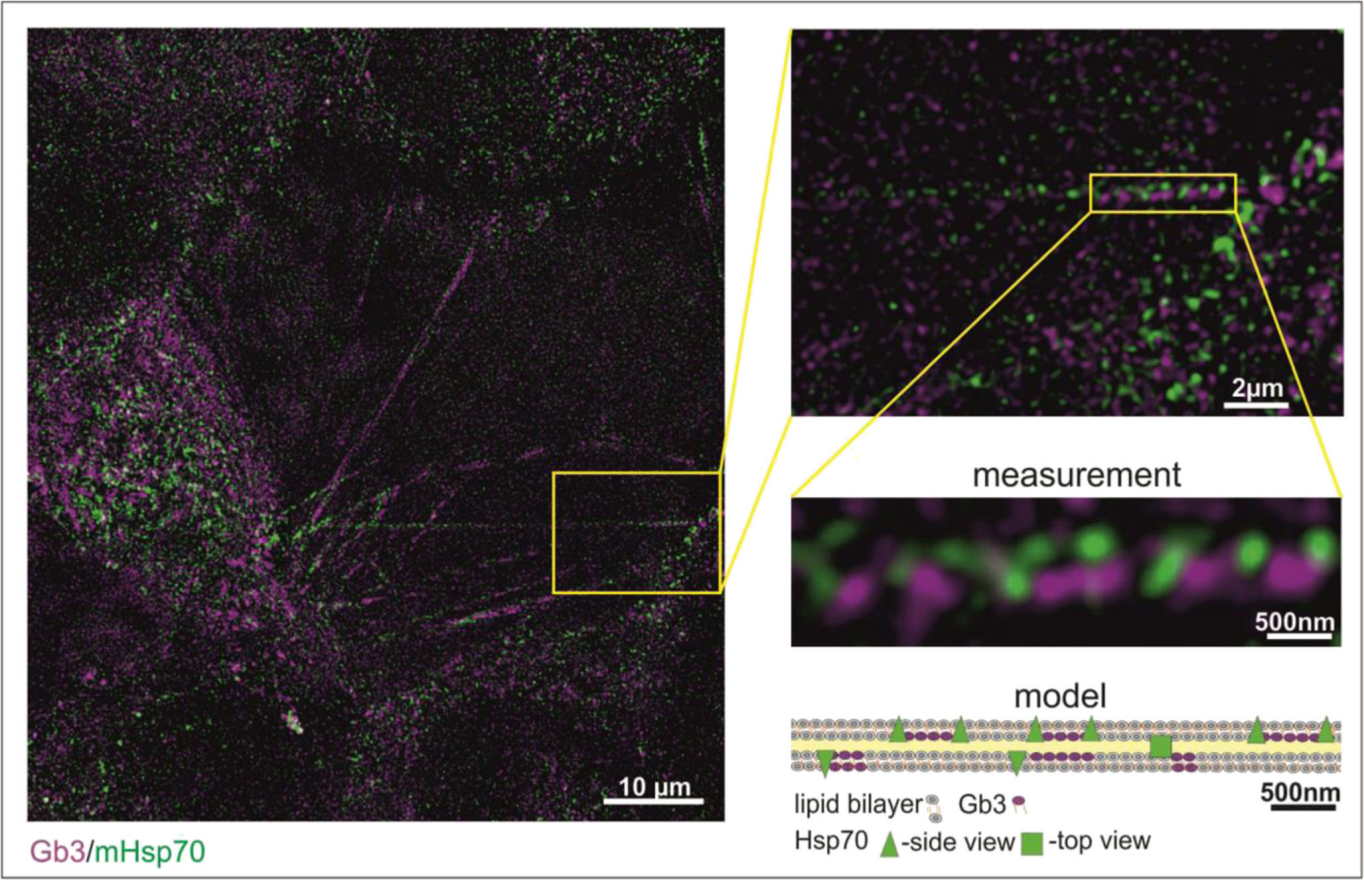
STED microscopy-based model of the organization of Gb3 (magenta) and Hsp70 (green) in TNTs at a × 13 imaging magnification

The acquired imaging data and the quantification of the TNTs allowed us to propose the following model that describes the structure of mHSP70 and Gb3 positive TNTs. Figure 4 shows a schematic representation of a total magnification of 130 (× 13 imaging magnification, × 100 objective magnification) of a representative region of a TNT in U87 cells. Although labelling of both structures is present throughout the whole TNT, no co-localization is visible within the resolution of 100 nm. Throughout the whole length of the TNT, Hsp70 indicated in green alternates with Gb3 in magenta and no overlapping regions are visible (no white regions). This clustering of Hsp70 and Gb3 is resembled also in cholesterol-rich microdomains of tumor cell membranes (Gehrmann et al. 2008).

### Quantification of TNT network connection types

**Fig. 5:**
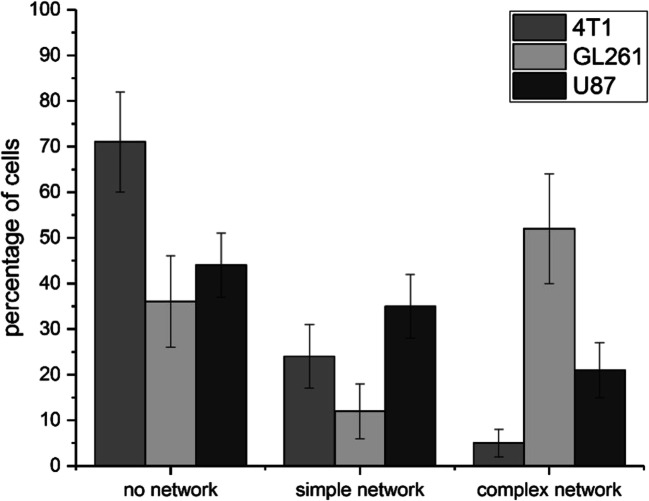
Characterization of the major three categories of nanostring network connections (no, simple, complex network connections) in 4T1 (dark gray), GL261 (light gray), and U87 (black) tumor cells

Although the absolute number of TNTs per cell differed drastically within one cell line, the type of network connections appeared to be specific for an individual cell line. The following three major connection categories could be distinguished: “no connecting networks,” “simple network connections” between two cells, and “complex network connections” between two or more cells. The different network types (no, simple, complex network connections) were quantified in the different tumor cell types (Fig. 5). The majority of 4T1 tumor cells (71 ± 12%) showed no network connections (*p* < 0.05, one-sided, unpaired *t* test), 24 ± 7% simple and 5 ± 3% of the 4T1 cells complex network connections. In contrast, GL261 cells predominantly revealed complex (52 ± 13%) network connections (*p* < 0.05, one-sided, unpaired *t* test), 12 ± 7% simple, and 36 ± 11% of the GL261 cells revealed no network connections, and 44 ± 7% of the U87 cells showed no, 35 ± 7% simple, and 20 ± 6% complex network connections.

## Discussion

In this study, the role of mHsp70 in the formation of cell-to-cell connections via TNTs was studied in three different tumor cell lines under physiological 2D cell culture conditions using super-resolution, live-cell STED nanoscopy. Due to technical limitations of the STED nanoscopy system, the measurements were performed at room temperature and not at 37 °C. These conditions are not perfect, but tumor cells stayed in the physiological culture medium alive during the whole experimental procedures. Further developments are ongoing to adopt the nanoscopy system to a temperature of 37 °C, 3D cell culture models, and animal models.

In general, Hsp70 was present in all TNTs of all three cell lines, as determined at a resolution of 100 nm. Hsp70 that clusters in TNTs originates from cholesterol-rich microdomains containing Hsp70, cholesterol, and Gb3. The measured diameter of Hsp70-based TNTs in different tumor cell types ranged between 120 and 240 nm which is comparable to that of published data showing an average diameter of 50 nm to 200 nm for nanotubes (Rustom et al. 2004). A more detailed analysis of the clustering of Hsp70 and Gb3 in living tumor cells revealed a close proximity of both markers in the plasma membrane Hsp70 (Multhoff and Hightower 2011) and TNTs. We therefore propose a model of the clustering of mHsp70 and Gb3 within the TNTs, which can be expanded to any other structures clustered at or in TNTs. This provides the first basis to understand the structural protein and lipid composition of TNTs.

Furthermore, we characterized the network formation complexity, which appeared to be cell-type specific. GL261 cells form more complex cellular networks than U87 and 4T1 tumor cells that also showed differences in their cell-to-cell communication networks. We hypothesize that the capacity of a tumor cell to form intracellular networks might impact on important hallmarks of cancer (Hanahan and Weinberg 2011) such as proliferation, migration, and invasiveness, which was also shown by Osswald et al. (2015). Therefore, in the future, live-cell imaging might provide a useful tool to characterize cell-to-cell communications among tumor cells or tumor cells with cells of the tumor microenvironment.

In contrast to previously published work (Marzo et al. 2012), the formation of Hsp70-based TNTs appears to be independent of environmental stress. Moreover, we could demonstrate that ionizing irradiation as a stress factor results in a complete loss of tumor-derived TNTs (Supplementary Figure 2). One might speculate that following stress, Hsp70 retranslocates from TNTs into the plasma membrane to stabilize the membrane. In line with this hypothesis, we have shown previously that the mHsp70 density increases after stress (Stangl et al. 2011; Murakami et al. 2015). Concomitantly, membrane-bound Hsp70 dissociates from the lipid raft component Gb3 and associates with the non-raft component phosphatidylserine (PS) (Schilling et al. 2009) that translocate from the inner to the outer plasma membrane leaflet. The elevated Hsp70 membrane expression density on tumor cells might be explained on the one hand by a stress-induced increase in the Hsp70 synthesis which results in an increased translocation of Hsp70 from the cytosol to PS in the plasma membrane, and on the other hand, by a fusion of Hsp70-containing nanotubes with the plasma membrane.

## Electronic supplementary material

ESM 1 (DOCX 1333 kb)
