# Supplementary material for: Membrane Hsp70-supported cell-to-cell connections via tunneling nanotubes revealed by live-cell STED nanoscopy
Source: Cell Stress Chaperones. 2019 Jan 10;24(1):213–21. doi: 10.1007/s12192-018-00958-w (PMC6363613; doi:10.1007/s12192-018-00958-w)
Supplement: Supplementary file 1 — (DOCX 1333 kb) [file 12192_2018_958_MOESM1_ESM.docx]

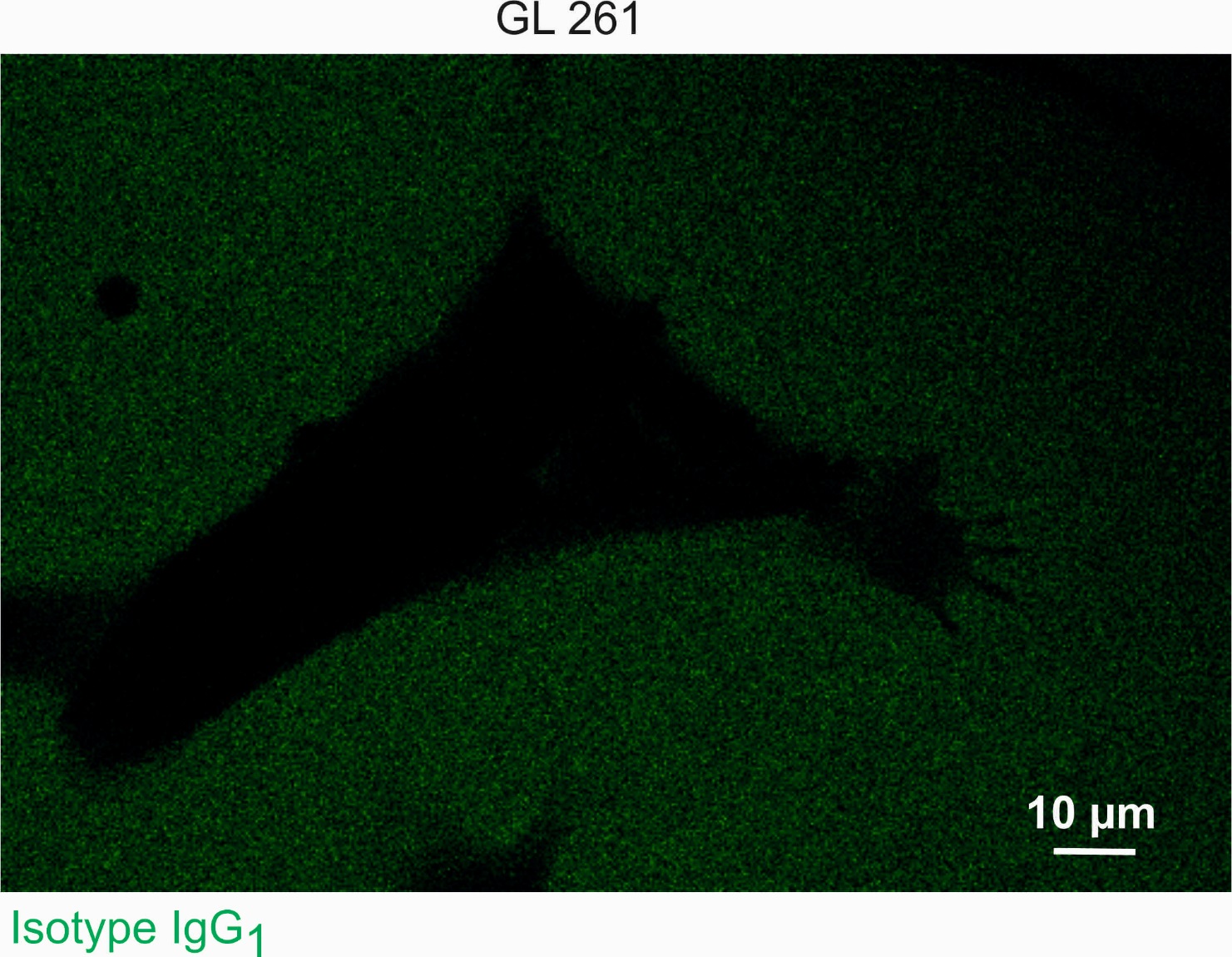


Supplementary Figure S1: GL261 cells stained with an IgG1 isotype matched FITC-conjugated control antibody. No specific cellular staining is visible.


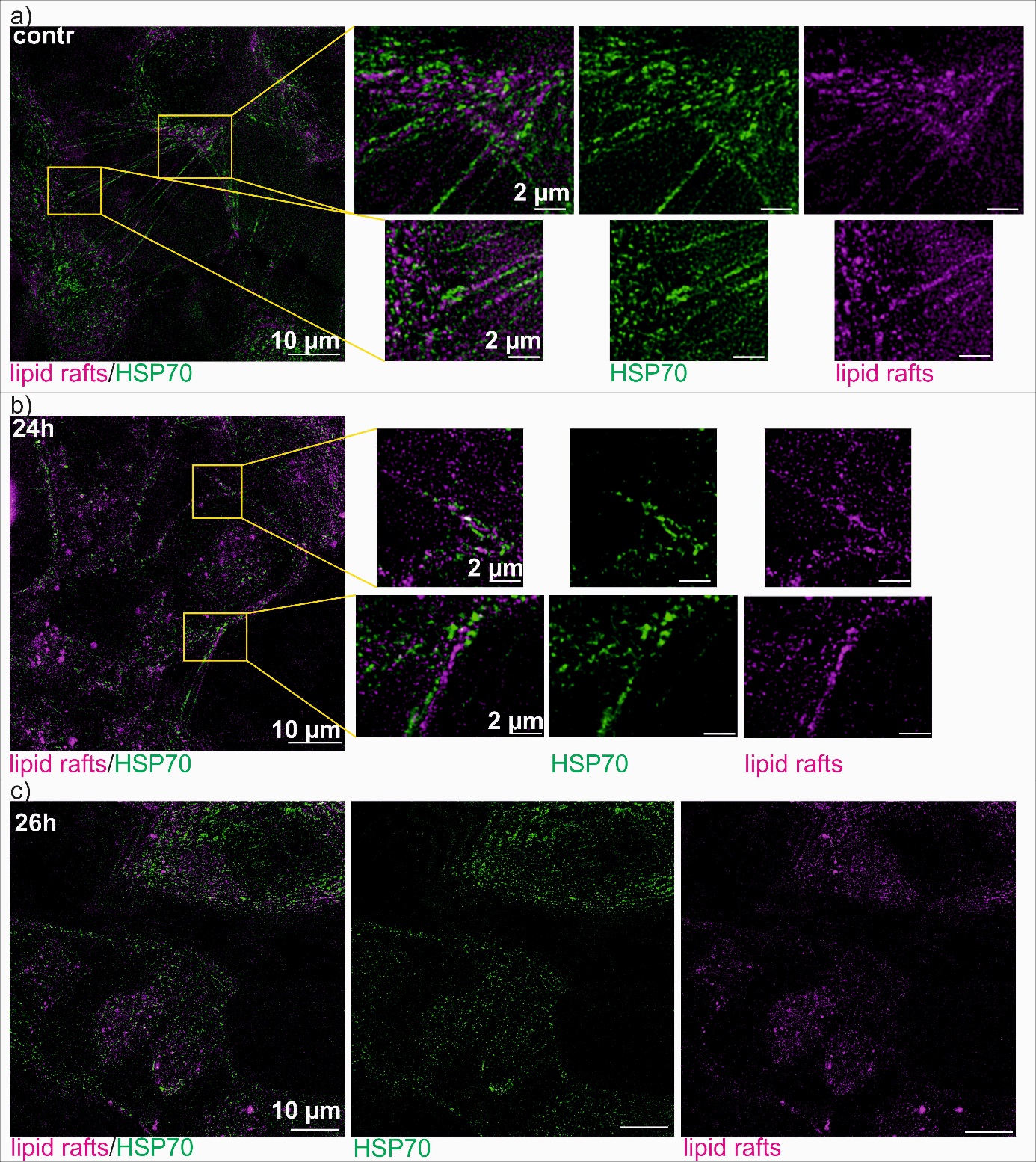


Supplementary Figure S2: A sublethal irradiation induces a drastic reduction of HSP70-containing nanotubes between U87 cells a) non-irradiated U87 cells. U87 cells 24 h (a) and 26 h (b) after irradiation with 0.5 Gy (2 MeV alpha-particles).
